# Enhancing post-traumatic stress disorder patient assessment: Leveraging Natural Language Processing for Research of Domain Criteria Identification using electronic medical records

**DOI:** 10.21203/rs.3.rs-3973337/v1

**Published:** 2024-02-21

**Authors:** Oshin Miranda, Sophie Kiehl, Xiguang Qi, Neal David Ryan, Levent Kirisci, M. Daniel Brannock, Thomas Kosten, Yanshan Wang, LiRong Wang

**Affiliations:** University of Pittsburgh; Colorado State University; University of Pittsburgh; University of Pittsburgh; University of Pittsburgh; RTI International; Baylor College of Medicine; University of Pittsburgh; University of Pittsburgh

**Keywords:** Post-traumatic stress disorder, research of domain criteria, real-world evidence, clinical notes, natural language processing

## Abstract

**Background:**

Extracting research of domain criteria (RDoC) from high-risk populations like those with post-traumatic stress disorder (PTSD) is crucial for positive mental health improvements and policy enhancements. The intricacies of collecting, integrating, and effectively leveraging clinical notes for this purpose introduce complexities.

**Methods:**

In our study, we created an NLP workflow to analyze electronic medical record (EMR) data, and identify and extract research of domain criteria using a pre-trained transformer-based natural language model, allmpnet-base-v2. We subsequently built dictionaries from 100,000 clinical notes and analyzed 5.67 million clinical notes from 38,807 PTSD patients from the University of Pittsburgh Medical Center. Subsequently, we showcased the significance of our approach by extracting and visualizing RDoC information in two use cases: (i) across multiple patient populations and (ii) throughout various disease trajectories.

**Results:**

The sentence transformer model demonstrated superior F1 macro scores across all RDoC domains, achieving the highest performance with a cosine similarity threshold value of 0.3. This ensured an F1 score of at least 80% across all RDoC domains. The study revealed consistent reductions in all six RDoC domains among PTSD patients after psychotherapy. Women had the highest abnormalities of sensorimotor systems, while veterans had the highest abnormalities of negative and positive valence systems. The domains following first diagnoses of PTSD were associated with heightened cue reactivity to trauma, suicide, alcohol, and substance consumption.

**Conclusions:**

The findings provide initial insights into RDoC functioning in different populations and disease trajectories. Natural language processing proves valuable for capturing real-time, context dependent RDoC instances from extensive clinical notes.

## Introduction

Post traumatic stress disorder (PTSD) is a prevalent mental health condition affecting 6.8–7.8% of the general US population, with higher rates in areas of civil unrest or armed conflict[1]. It is characterized by disabling symptoms that often persist, leading to significant impairment in economic and social functioning and an increased risk of mortality[2]. The disorder, as defined by DSM-5, encompasses symptom clusters such as hyperarousal, persistent re-experiencing of trauma, avoidance of trauma-related stimuli, and negative alterations in cognition and mood. Empirical findings reveal heterogeneity within PTSD, with subtypes characterized by hyperarousal or dissociation[3]. Untreated PTSD patients face an elevated risk of suicide-related events, substance use disorders, and other neuropsychiatric disorders.

Comorbidities exacerbate challenges, compromising social adjustments, treatment outcomes, and increasing the likelihood of early treatment termination[4]. Current approaches involve targeting individual conditions either concurrently or sequentially due to a lack of specific evidence-based interventions. Despite some interventions showing effectiveness, a substantial proportion of individuals with PTSD experience limited improvement, necessitating exploration of new, theory-based treatment options[5].

Aligning with the National Institute of Mental Health’s (NIMH) research initiatives, a promising approach is to comprehend comorbidities through shared functional domains or transdiagnostic factors. Despite efforts towards transdiagnostic treatment, the Diagnostic and Statistical Manual of Mental Disorders (DSM-5) continues to use a categorical approach, potentially limiting treatment efficacy[6]. The Research Domain Criteria (RDoC) project, launched by the NIMH, presents a novel research framework aiming to overcome these limitations by identifying transdiagnostic mechanisms of mental disorders[7].

Disruptions in economic and social behaviors are significant sequelae of PTSD, leading to marital and parenting problems, high rates of comorbid disorders, unemployment, homelessness, and imprisonment[8]. The relationship between PTSD and comorbid disorders is complex and bidirectional. Traumatic stress is recognized as a vulnerability factor for substance use disorder (SUD), with a substantial proportion of SUD patients meeting PTSD criteria[9].

Recent research indicates that PTSD is linked to a diverse range of multimodal risk factors[10]. Predictive methods for PTSD using electronic medical records (EMR) data need to accommodate various combinations of risk indicators. The application of computational methods and machine learning to health data holds promise for advancing our understanding of health conditions. This study aims to characterize PTSD by leveraging the knowledge of the RDoC framework using unstructured EMR data (e.g., clinical notes). Clinical notes are crucial in healthcare, providing a detailed patient narrative that goes beyond structured data to capture nuanced information. They ensure continuity of care by documenting the patient’s journey over time, enabling tracking of conditions and adjustments to treatment plans. These notes offer insights into disease patterns and treatment effectiveness. They aid in diagnostics by capturing subjective and objective symptom information, assisting in accurate diagnoses and treatment planning. Additionally, clinical notes consider both biomedical and psychosocial factors, which is essential in addressing each patient’s unique needs and preferences. The novel framework incorporates keyword dictionaries, context-specific sentence dictionaries, the application of sentence transformer model, and Identification of RDoC domains in two distinct use cases. By leveraging unstructured “big data,” this research represents a crucial step towards integrating the RDoC framework into treatment research for comorbid conditions, offering insights into etiology and treatment responses.

## Methods

### Data source:

We obtained data from the Neptune system which is a clinical data warehouse at UPMC (January 2004 – October 2020). The database includes demographics, diagnoses, prescriptions, and test results. This study utilized 5.67 million clinical notes from PTSD patients as identified by ICD9/10 codes (refer to Supplementary information: Appendix A for details)[11, 12].

### Building of Dictionaries:

We summarized the current research on PTSD integration into the RDoC framework and built our context-dependent keyword and sentence dictionary from that research and subject matter experts (SME). Artificial intelligence models struggle to classify narratives in niche domains when they have not been trained on them or tailored to the specialized subject matter. We aim to address this problem by including SMEs in dictionary development[13]. Our dictionaries include the following attributes:

#### Negative Valence Systems:

Research consistently supports the relevance of negative valence systems in PTSD, characterized by fear and anxiety symptoms, and particularly anxious avoidance of trauma-related cues. This anxiety may generalize to neutral cues during flashbacks, and key mechanisms involving the amygdala, prefrontal cortex, and hippocampus are implicated in fear conditioning and extinction[14], [15]. PTSD is associated with overgeneralized fear, impairments in fear extinction, and cue generalization. Dysfunctional amygdala and hypoactivity in the ventromedial prefrontal cortex contribute to heightened fear responses and hindered extinction[16]. Genetic factors, including the BDNF val66met-allele, are linked to impaired fear extinction, impacting treatment response[17].

#### Positive Valence Systems:

Positive valence systems, focusing on reward learning and valuation, are understudied in PTSD, with anhedonia reflecting emotional numbing and diminished goal-oriented behavior[18]. Reward processing deficits involve dopamine and serotonin systems, influenced by genetic factors[19]. Oxytocin and SSRIs show promise in addressing reward deficits and anhedonia in PTSD treatment[20].

#### Cognitive Systems:

Cognitive deficits in PTSD affect attention, planning, and memory, with attentional bias towards threat stimuli and memory biases contributing to hyperarousal[21]. Epigenetic modifications and gene polymorphisms, like in the glucocorticoid receptor (GR) gene, are linked to memory deficits in PTSD[22], [23]. Effective treatment may improve cognitive deficits.

#### Arousal and Regulatory Systems:

Hyperarousal, a core symptom of PTSD, involves heightened nervousness, sleep problems, and increased startle responses, with sympathetic nervous system overdrive contributing[8], [24], [25]. Genetic variations in adrenergic receptors influence emotional memory, and medications like prazosin and propranolol show efficacy in treating PTSD-related hyperarousal[26], [27].

#### Systems for Social Processes:

Social processes, including attachment, communication, and self-perception, are affected in PTSD, particularly in cases of complex PTSD or interpersonal trauma[28]. Concepts like shame, guilt, and paranoid distrust are prevalent in interpersonally traumatized PTSD patients and merit further study[29], [30], [31].

#### Sensorimotor Systems:

Current transdiagnostic research explores sensorimotor abnormalities in children, individuals at risk of psychosis, and first-episode psychosis patients, among others[32], [33], [34]. Sensorimotor dysfunction, recognized only in recent years, can be used to enhance early Identification and develop effective treatments.

**Figure 1:**
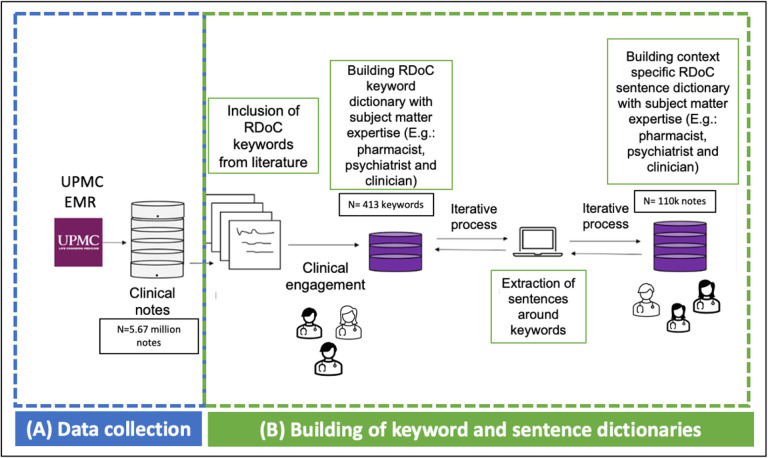
Iterative flow of our sentence dictionary development during ad-hoc collection, clinical engagement, and data integration

**Figure 2:**
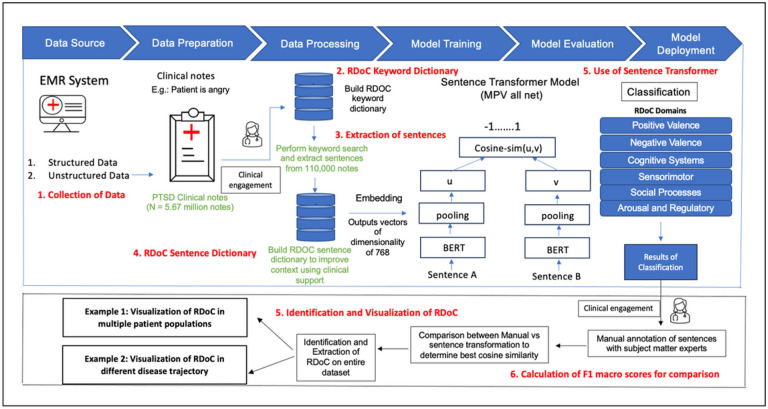
Diagrammatic representation of workflow of our study

We utilized a “Human-in-the-Loop” approach, incorporating subject matter expertise to develop sentence dictionaries [35]. We developed sentence dictionaries to address the limitation of existing keyword dictionaries, which often include common English words lacking context specificity. Through sentence dictionaries, we aim to encompass the entire context in which a word associated with RDoC domain is utilized. Figures 1 and 2 illustrate the iterative flow of sentence dictionary development and the study workflow, respectively. Supplementary tables 1 and 2 depict the RDoC keyword and sentence dictionaries. The study aims to identify population and disease-trajectory-specific RDoC domains for early PTSD diagnosis and treatment research.

The steps of the iterative workflow correspond to. (A) Data collection (B) Building of keyword and sentence dictionary from literature[36], [37] and SMEs

### Sentence transformer model:

We used the pre-trained model all-mpnet-base-v2 [38], a transformer-based natural language model to identify the presence of RDoC domains in clinical notes of PTSD patients. The model is based on the MPNet architecture and has the highest performance in generating sentence embeddings according to Sentence-Transformers [38], [13]. We did not perform any additional fine-tuning on our dataset. The model that was provided by Sentence-Transformers was used in its original form, which has an output dimension of 768. Thus, the output of this model for each RDoC in the PTSD dataset is an embedding that has a length of 768.

The RDoC extraction pipeline involves: 1. Collecting and preprocessing of 5.67 million PTSD clinical notes from the UPMC EMR system. 2. Building of a keyword dictionary from literature and SMEs. 3. Conducting keyword searches and extracting sentences from 110,000 clinical notes. 4. Creating a context-specific RDoC sentence dictionary, evaluated by SMEs for categorization and inclusion (Table 3). 5. Utilizing sentence transformers to extract RDoC information by comparing cosine similarity scores between sentences, where Sentence A is the new sentence from a clinical note that is being annotated and Sentence B is the sentence from the sentence dictionary. The selection criteria involve identifying the presence of a keyword in both sentences. Once the keyword is identified, the sentence transformer focuses on only those sentences where the keyword is present in sentence B. Subsequently, the transformer assesses the cosine similarity scores between Sentence A and each potential Sentence B until the optimal match is determined. This is a supervised approach. 6. Calculating F1 macro scores to determine the optimal threshold for identifying RDoC categories, comparing manual annotation by SMEs with sentence transformer. (7) Identifying and Visualizing RDoC in two use cases (i) Across multiple patient populations and (ii) Throughout various disease trajectories.

### Statistical analysis:

The delineation of RDoC domain categories for data collection underwent an iterative refinement due to the extensive volume of clinical notes, as illustrated in Fig. 2. Initial stages encompassed employing a keyword dictionary, extracting sentences with these keywords, labeling sentences into categories through SMEs, and leveraging sentence transformers for RDoC domain Identification along with corresponding keywords. Table 3 shows the count of identified patients, with SMEs reviewing a subset of randomly selected cases (N = 8,351 sentences). These procedures were crucial in the labeling process.

SMEs initially developed labels that best defined each domain, but noticed missing keywords, prompting ongoing refinement. Inclusion of these keywords enhanced the dictionary, improving RDoC Identification accuracy. In the final iteration, SMEs verified correct assignments. Comparing manual SME annotations with sentence transformer results, Supplementary Table 3 shows that a 0.3 cosine similarity threshold yielded the best F1 macro scores across all RDoC domains, ensuring an F1 score of at least 80% across all domains (**N = 8,351 sentences**).

## Results

Baseline information, encompassing 12 categories of mental disorders (see Appendix B) [39], age, gender, and the follow up times, is detailed in Table 1. Following the data processing, dictionary development, and application of the sentence transformer (Steps 1–6) as seen in Fig. 2, we identified the Top 10 keywords and their respective counts identified in our dataset (Supplementary table 4), and the top keyword along with an example corresponding sentence in our dataset (Supplementary table 5).

In mental health research, especially in PTSD, innovative approaches are essential to address gaps in understanding the condition’s complex mechanisms. Traditional methodologies may overlook crucial details in clinical narratives, hindering the Identification of nuanced patterns and tailoring interventions. Leveraging the sentence transformer model, we explored PTSD-related information more comprehensively in unstructured data. Emphasizing the crucial role of RDoC Identification, two compelling use cases illustrate the potential of this approach:

### Identification of RDoC domains across different patient populations

**Figure 3:**
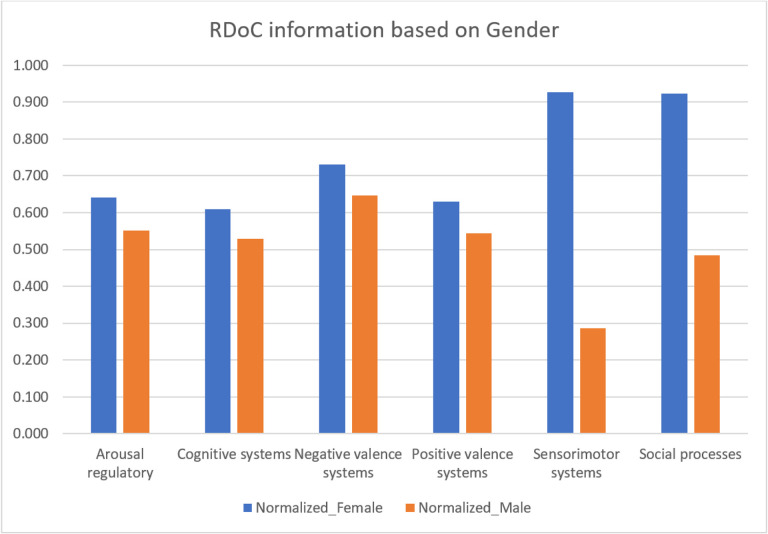
RDoC domains across different patient populations: Male and Female

Our goal is to empower clinicians and researchers by understanding how various RDoC domains manifest in different patient populations, facilitating the development of targeted strategies for unique needs. Analyzing demographic data from structured EMRs, we found females (N = 14,862) exhibited a higher prevalence of RDoC information than males (N = 7,333) (Fig. 3 and Supplementary table 6) with a statistical significance of P = 0.03125. Abnormal sensorimotor occurrences, potentially linked to prepulse inhibition (PPI), were more prominent in females. PPI, a measure of sensorimotor filtering, has been associated with conditions like challenges in suppressing sensory or motor information. A study found that trauma-exposed women with PTSD exhibited deficits in PPI, supporting our empirical findings of sensorimotor abnormalities in individuals with PTSD[40].

**Figure 4:**
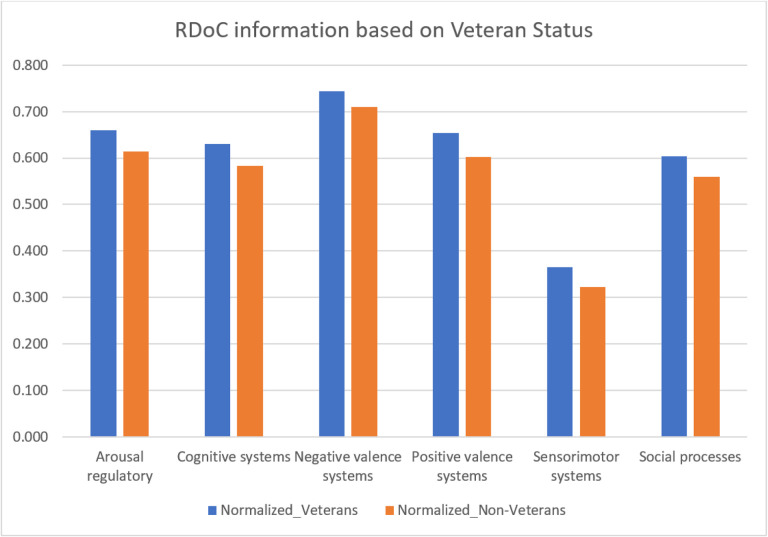
RDoC domains across different patient populations: Veterans and Non-Veterans

We analyzed veteran status and psychotherapy details from unstructured EMRs. And explored differences in behaviors, cognitions, and mental health symptoms between veterans (N = 4,657) and non-veterans (N = 17,114) (Fig. 4 and Supplementary table 7), finding the instances of negative valence system constructs, including acute threat (e.g., fear, panic), potential threat (e.g., inhibition, worry), sustained threat (e.g., chronic stress), frustrative non-reward (e.g., reactive aggression), reduced behavioral activation (e.g., anhedonia), and loss (e.g., low well-being), and positive valence system constructs including reward seeking and consummatory behaviors in veterans. Our results were statistically significant with a p-value of 0.03552. This aligns with findings from another study[41], suggesting the need for veteran-specific RDoC markers, though further research is warranted.

**Figure 6:**
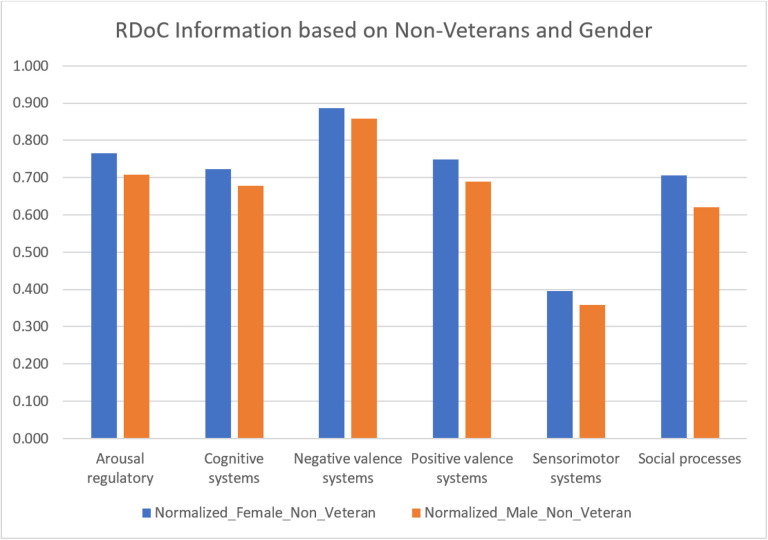
RDoC domains across different patient populations: PTSD patients based on gender and non-veteran status

**Figure 7:**
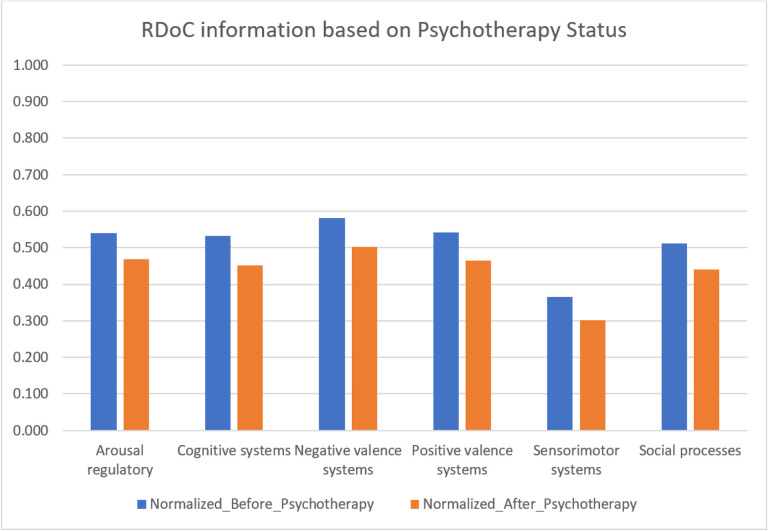
RDoC domains across different patient populations: PTSD patients before psychotherapy and after Psychotherapy

We conducted a systematic examination of RDoC domains among PTSD patients, incorporating gender and veteran status (refer to Fig. 6, Fig. 7, and Supplementary Tables 8 and 9), the distinct groups include female veterans (N = 2766), male veterans (N = 2855), female non-veterans (N = 13,775), and male non-veterans (N = 5228). The significant findings (P = 0.03125 for both comparisons) revealed fewer occurrences across all RDoC domains in male PTSD patients compared to their female counterparts, a trend persisting even when considering veteran status. This observation highlights potential gender-related differences, extending across both the veteran and non-veteran populations.

We investigated the differences RDoC domain in PTSD patients before psychotherapy (N = 2,262) and after psychotherapy (N = 3,189) (Fig. 7 and Supplementary table 10). The significant results (p value = 0.03125) revealed a consistent decrease in instances across all RDoC domains in PTSD patients after psychotherapy. This observation highlights the potential positive impact of psychotherapy on alleviating symptoms associated with PTSD. While further investigations are necessary, our findings contribute to advancing PTSD research by shedding a direct positive light on the potential impact of psychotherapy on various RDoC domains.

### Example 2: Impact of RDoC on overall disease trajectory

**Figure 8:**
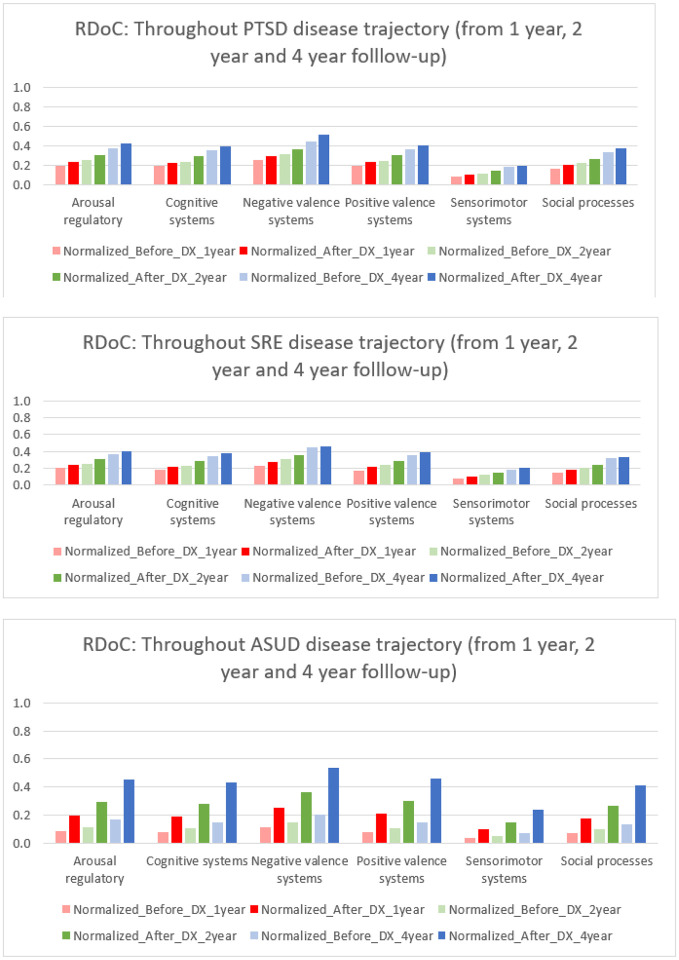
Overall RDoC changes throughout Disease Trajectory (PTSD, SRE, ASUD)

Exploring RDoC domains in the trajectory of PTSD yields valuable insights into the dynamic nature of mental health conditions, allowing researchers to identify critical phases in progression and understand underlying mechanisms. This knowledge is pivotal for developing targeted interventions at specific disease stages, potentially preventing exacerbation, and guiding effective interventions. In our analysis, we identified PTSD patients with first diagnoses of PTSD (N = 22,198), suicide-related events (SREs) (N = 5,590), and alcohol and substance use disorder (ASUD) (N = 13,993) within 1, 2, and 4 years of follow-up (Fig. 8 and Supplementary tables 11–19) with the p values of 0.00001475. RDoC information extracted from unstructured EMR data revealed higher instances of overall RDoC domain changes in SRE-diagnosed patients compared to PTSD-diagnosed patients. Patients with ASUD diagnoses exhibited a substantial increase in RDoC domains, particularly in negative and positive valence systems, over the 4-year follow-up, emphasizing the importance of RDoC Identification in studying PTSD sequelae. Further research is needed to comprehend the relationship between negative valence systems and ASUD.

Collectively, these findings highlights the positive impact of identifying RDoC symptoms in different populations and diagnoses. Significantly, these results propose potential advantages in the development and utilization of measures focusing on transdiagnostic factors aligned with the RDoC.

### World Cloud:

**Figure 9:**
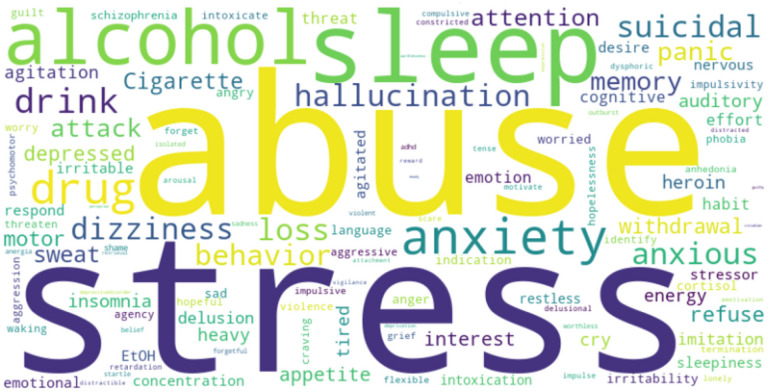
RDoC Keywords for PTSD patients

Word clouds visually represent qualitative data, with word size reflecting frequency or significance. Applied in medical literature and beyond, they creatively highlight patterns[42]. Our assessment explored RDoC’s impact on PTSD, using Python (v3.8.8) to generate word clouds. Figure 9 illustrates the prominence of RDoC keywords (e.g., stress, abuse, alcohol, sleep, suicidal, anxiety, hallucination, and attention) using all the clinical notes of the PTSD population, transcending specific domains. Supplementary information details each keyword within its RDoC domain (Supplementary Figs. 1–6),.

The word cloud was crafted from the clinical notes of PTSD patients, capturing insights related to RDoC domains. The magnitude of each word is directly linked to the number of instances during the analysis.

## Discussion

Our study delves into PTSD within the RDoC framework, exploring if multimodal patient information aligns with RDoC domains. It uniquely examines functioning RDoC domain alterations in PTSD patients across diverse populations. Utilizing custom dictionaries, RDoC was identified as six domains (Cognitive, Positive Valence, Negative Valence, Social Processes, Sensorimotor, and Arousal/Regulatory Systems). The study’s approach, leveraging existing measures, demonstrates promise in utilizing Artificial intelligence tools for context-specific RDoC domain Identification in neuropsychiatric research and promotes integration of research discoveries to offer a potentially valuable dimensional perspective on patients diagnosed with PTSD.

In line with our hypotheses, targeted domains increased in severity post-PTSD diagnosis, consistent with findings by Coffey et al indicating a general shift in symptoms across specific diagnostic categories including substance use, depression, and anxiety[43], [44]. Our approach aligns with RDoC’s goal of uncovering underlying psychopathological mechanisms, departing from traditional categories to consider multimodal information for personalized treatment[45]. Analyzing pre-diagnosis values, we observed a rise in RDoC symptoms in patients diagnosed two years later compared to two years before PTSD diagnosis, over a 4-year follow-up. Lower RDoC domain scores were associated with reduced distress, sadness, aggression, worry, anhedonia, alcohol, and substance cravings. Conceptually grouping these symptoms within functioning domains, we identified associations such as distress and sadness with the arousal and regulatory system, aggression with social processing, anhedonia with cognitive system, worry with negative valence system, and alcohol and substance craving with the positive valence system, assessing overall domain functioning in these patients.

The Cognitive systems domain showed a reduced number of instances in PTSD patients after psychotherapy, aligning with the notion that subjective distress, uncontrollability, and unpredictability are key anxiety components[46]. Veterans, women, and those before psychotherapy exhibited higher severity. Psychotherapy, addressing dysfunctional trauma-related cognitions, led to beneficial reductions in emotional distress, emphasizing the interplay between cognitive and affective systems. Further cognitive-specific therapies may enhance cognitive restructuring[47]. Negative Valence Systems instances peaked in PTSD patients before psychotherapy but decreased after psychotherapy, linked to reduced subjective distress and alcohol cravings[48]. This highlights the RDoC domain’s role in PTSD outcomes. Positive Valence systems showed higher instances in veterans and PTSD patients before psychotherapy, suggesting reward system dysfunction[18]. Trauma-related abnormalities in neural structure contribute to symptoms like anhedonia and reduced motivation[49]. PTSD, more prevalent in women, involves dysregulated neuronal, hormonal, and immune mechanisms, impacting sensory processing and sensorimotor systems[50]. Social cognition encompasses processes linking the perception of social information to behavioral responses, including perception, attention, decision-making, memory, and emotion[51]. Social processing deficits such as fear responses associated with trauma may be alleviated by psychotherapy, emphasizing its positive effect. Arousal/Regulatory domain scores decreased in PTSD patients after psychotherapy, highlighting the significance of physiological symptoms. Sleep patterns impact distress and the Arousal/Regulatory domain, emphasizing their role in PTSD development[52].

Our research is pivotal in advancing PTSD studies by employing natural language processing techniques to extract RDoC information from unstructured EMR data. This approach enhances our understanding of subtle variations in symptomatology, contributing to a more comprehensive view of PTSD. Additionally, our study highlights the importance of personalized and context-specific mental health interventions. Identifying RDoC domains allows tailored interventions for specific patient populations and disease stages, addressing unique challenges. This personalized approach holds the potential to significantly enhance treatment outcomes and patient well-being. Overall, our research not only addresses critical gaps in PTSD research methodology but also informs policy decisions and engages stakeholders, ensuring the translation of RDoC Identification benefits into meaningful advancements in mental healthcare.

### Limitations:

Our study has notable limitations that warrant consideration. In constructing domains aligned with RDoC, we repurposed words from existing measures due to the absence of validated measures explicitly designed for RDoC domains. While our use of Artificial intelligence introduces potential measurement error, the validation by SMEs demonstrated satisfactory results. Data constraints from UPMC, limited to routine medical notes, excluded special mental health reports hindering the incorporation of RDoC measures and further validation. Our focus on five out of the six RDoC domains, particularly in the context of PTSD, raises questions about the applicability of the sixth domain (Sensorimotor Systems). Additionally, the study’s scope is confined to PTSD, and the specific impacts of various treatments or substance use on observed changes remain unclear. Despite these limitations, our study contributes to RDoC’s short term objectives by pioneering measurement tools for its domains, offering valuable insights for future assessments within the framework.

### Future Study:

Considering both strengths and limitations, future research should focus on developing valid and reliable measures for RDoC domains, aligning with the RDoC matrix’s comprehensive, multi-unit analysis. Integrating RDoC data into deep-learning models predicting adverse events (e.g. substance use disorder, opioid use disorder, suicide related events) could enhance measure formulation. Expanding the investigation to cover all six RDoC domains is crucial, allowing exploration of associations between specific functioning domains and more pronounced changes with multimodal information. This insight can refine therapeutic change mechanisms, guiding the development of targeted treatments. Future research should extend beyond PTSD, including diverse populations and treatments, enhancing generalizability, and understanding the applicability of RDoC domains across various clinical contexts.

## Conclusion

This paper delves into a crucial aspect of the ever-expanding significance of RDoC as interventions aimed at leveraging multi-modal real-world data continue to progress. We present a systematic approach for obtaining high-quality data from longitudinally collected clinical notes. Our outlined process encompasses data extraction, acquisition, and preparation for analysis, utilizing a transformer-based model that helps identify RDoC in a context-specific manner. Given the escalating importance of RDoC in the field of neuropsychiatric research, our procedure along with the use cases discussed in this paper hold substantial potential for broad applicability across diverse clinical settings and populations.

## Figures and Tables

**Figure 5:**
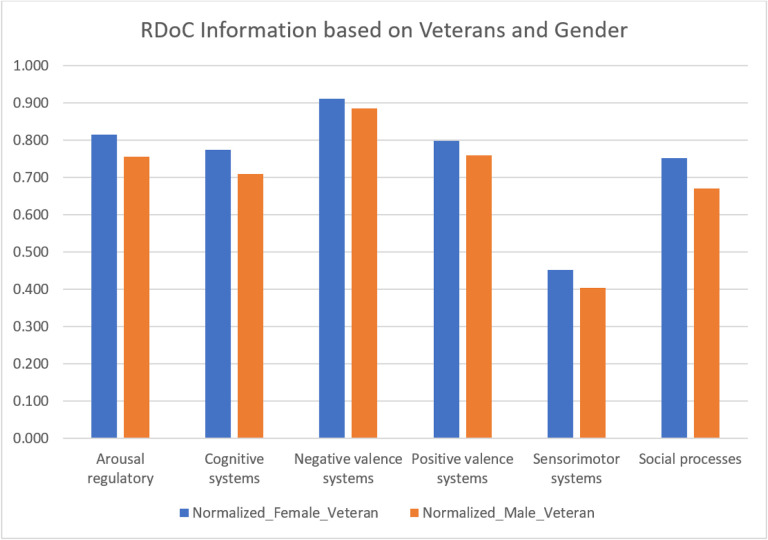
RDoC domains across different patient populations: PTSD patients based on gender and veteran status

**Table 1:** Number of patients identified by the Sentence Transformer and number of randomly selected cases to review and manually annotated by subject matter experts

RDoC	No. of Patient cases	Randomly selected cases to review and manually annotated by Subject matter experts
Arousal regulation	18724	443
Cognitive systems	17740	2165
Negative valence	21829	2629
Positive valence	18360	2797
Sensorimotor systems	9770	74
Social process	17014	243

**Table 2:** Baseline characteristics of the cohort:

	Overall
**n**	38801
**GENDER (%)**	
**FEMALE**	24501 (63.1)
**MALE**	14294 (36.8)
**UNKNOWN**	6 ( 0.0)
**Age (mean (SD))**	37.81 (16.83)
**RACE (%)**	
**ALASKA NATIVE**	7 ( 0.0)
**AMERICAN INDIAN**	179 ( 0.5)
**BLACK**	7044 (18.2)
**CHINESE**	26 ( 0.1)
**DECLINED**	299 ( 0.8)
**FILIPINO**	23 ( 0.1)
**GUAM/CHAMORRO**	3 ( 0.0)
**HAWAIIAN**	9 ( 0.0)
**INDIAN (ASIAN)**	50 ( 0.1)
**JAPANESE**	6 ( 0.0)
**KOREAN**	16 ( 0.0)
**NOT SPECIFIED**	514 ( 1.3)
**OTHER ASIAN**	108 ( 0.3)
**OTHER PACIFIC ISLANDER**	25 ( 0.1)
**SAMOAN**	2 ( 0.0)
**UNKNOWN**	322 ( 0.8)
**VIETNAMESE**	7 ( 0.0)
**WHITE**	30161 (77.7)
**Follow_up_time (mean (SD))**	4.44 (5.71)
**Cat1_1Year = 1 (%)**	4660 (12.0)
**Cat2_1Year = 1 (%)**	872 ( 2.2)
**Cat3_1Year = 1 (%)**	14378 (37.1)
**Cat4_1Year = 1 (%)**	540 ( 1.4)
**Cat5_1Year = 1 (%)**	11560 (29.8)
**Cat6_1Year = 1 (%)**	861 ( 2.2)
**Cat7_1Year = 1 (%)**	57 ( 0.1)
**Cat8_1Year = 1 (%)**	89 ( 0.2)
**Cat9_1Year = 1 (%)**	940 ( 2.4)
**Cat10_1Year = 1 (%)**	854 ( 2.2)
**Cat11_1Year = 1 (%)**	2293 ( 5.9)
**Cat12_1Year = 1 (%)**	172 ( 0.4)

## Data Availability

The data used in this study were from UPMC under a data use agreement. The authors are not permitted to distribute the data to any third party, but researchers may contact UPMC for data access.
